# Oxidative damage and chemokine production dominate days before immune cell infiltration and EAE disease debut

**DOI:** 10.1186/s12974-016-0707-3

**Published:** 2016-09-15

**Authors:** Henrik Hasseldam, Rune Skovgaard Rasmussen, Flemming Fryd Johansen

**Affiliations:** Department of Biomedical Sciences, Faculty of Health, University of Copenhagen, Ole Maaloes vej 5, DK-2200 Copenhagen, Denmark

**Keywords:** Experimental autoimmune encephalomyelitis, Neuronal degeneration, Chemokines, Reactive oxygen species, CNS inflammation

## Abstract

**Background:**

Multiple sclerosis is widely accepted as an inflammatory disease. However, studies indicate that degenerative processes in the CNS occur prior to inflammation. In the widely used animal model experimental autoimmune encephalomyelitis (EAE), we investigated the significance of degenerative processes from mitochondrial membrane potentials, reactive oxidative species, cell death markers, chemokines, and inflammatory cell types in brain, spinal cord, and optic nerve tissue during the effector phase of the disease, before clinical disease was evident.

**Methods:**

Sixty-two rats were placed in eight groups, *n* = 6 to 10. Four groups were immunized with spinal cord homogenate emulsified in complete Freund’s adjuvant (one served as EAE group), three groups were immunized with complete Freund’s adjuvant only, and a control group was injected with phosphate buffered saline only. Groups were sacrificed 3, 5, 7, or 12–13 days after the intervention and analyzed for early signs of CNS degeneration.

**Results:**

Loss of mitochondrial membrane potential and oxidative changes was observed days before clinical disease debut at day 9.75 ± 0.89. The early mitochondrial changes were not associated with cytochrome C release, cleavage of caspases 9 (38/40 kDa) and 3 (17/19 kDa), and cleavage of PARP (89 kDa) or spectrin (120/150 kDa), and apoptosis was not initiated. Axonal degeneration was only present at disease onset. Increases in a range of cytokines and chemokines were observed systemically as a consequence of immunization with complete Freund’s adjuvant, whereas the encephalitogenic emulsion induced an upregulation of the chemokines *Ccl2*, *Ccl20*, and *Cxcl1*, specifically in brain tissue, 7 days after immunization.

**Conclusion:**

Five to seven days after immunization, subtle decreases in the mitochondrial membrane potential and an increased reactive oxygen species burden in brain tissue were observed. No cell death was detected at these time-points, but a specific expression pattern of chemokines indicates activity in the CNS, several days before clinical disease debut.

## Background

Multiple sclerosis (MS) is widely accepted as an inflammatory disease with a primary autoimmune component. The immune system plays a central role in the pathogenesis of the disease but the etiology remains unknown [1]. In the clinic and according to the Revised McDonald Criteria, a diagnosis of MS may be supported by optical coherence tomography to detect early optic neuritis, magnetic resonance imaging for observation of multiple central nervous system (CNS) areas with neuronal damage, a cerebrospinal fluid analysis to investigate presence of non-specific inflammation markers, and visual evoked potentials to examine brain processing speed, although ultimately the diagnosis is a clinical diagnosis [2]. High doses of intravenous corticosteroids like methylprednisolone may be used for short-term treatment of symptoms, while long-term treatment include interferon (beta-1b or 1a), glatiramer acetate, natalizumab, and newer therapeutics like dimethylfumarate, teriflunomide, and fingolimod [3]. Common to these long-term treatment regimens is that the earlier they are administered to the patients, the more effective they are. Thus, early diagnosis is paramount in order to provide optimal treatment, thereby implicating a need to establish reliable translational experimental models for MS disease induction.

Although experimental autoimmune encephalomyelitis (EAE) is the most frequently used model for MS, EAE has several caveats compared to MS. One major difference is that EAE is induced by active immunization with CNS antigens, whereas the reason behind MS seems to be an interplay between genetic and environmental factors [4]. In addition, plaque composition and inflammatory cell types also differ between EAE and MS. Whereas EAE is driven primarily by CD4^+^ T cells, MS infiltrates also contain a high number of CD8^+^ T cells [5]. Investigators should be cautious of assuming a direct parallel when comparing EAE and MS.

Because EAE is an active immunization with myelin antigens, the general dogma is that disease induction requires the presence of activated encephalitogenic T cells in the CNS. Thus, it is puzzling that several studies have shown neural degeneration and death at stages in disease development where no inflammatory cells are present in the CNS. Investigators have found apoptosis in retinal ganglion cells as early as 5 days after immunization [6], whereas others report that mitochondria-derived oxidative stress 3 days after immunization results in oligodendrocytic and neuronal apoptosis [7]. Furthermore, administration of the anti-oxidant SOD2 has been shown to reduce tissue damage significantly, suggesting a causal relationship between oxidative stress and neural cell death [8]. In addition, axonal degeneration and loss has been shown to occur 7 days post-immunization, several days prior to inflammatory infiltration, myelin destruction, and clinical disease debut [9, 10]. Taken together, these papers indicate that axonal loss in EAE may precede inflammation and demyelination, partially mediated by oxidative stress. Providing further light to the etiology of multiple sclerosis, recent findings indicate that blood-brain barrier disruption may be one of the earliest symptoms leading to MS, where fibrinogen enters the CNS and leads to formation of insoluble fibrin, increased thrombin activity, activation of innate immunity, and inflammatory demyelination [11–13].

In order to further investigate and systematically characterize events prior to inflammation and demyelination, we induced EAE in the Dark Agouti rat strain using spinal cord homogenate emulsified in complete Freund’s adjuvant (CFA) resulting in 100 % disease incidence. The brain, spinal cord, cerebrospinal fluid, and peripheral blood were isolated after immunization and analyzed for early signs of degeneration in the form of oxidative damage, neuronal and glial apoptosis and necrosis, and cytokine and chemokine production, as well as inflammatory infiltration.

## Methods

### Animals

Female Dark Agouti rats (10–12 weeks old, Harlan, Boxmeer, The Netherlands) were housed three per cage in conventional cages with free access to food and water on 12-h light/dark cyclus. All animal procedures were conducted in accordance with the guidelines of the Danish Animal Experiments Committee (#2012-DY-2934-00001) and the Department of Experimental Medicine, University of Copenhagen.

### EAE induction

In order to induce EAE, the animals were immunized by subcutaneously injecting 150 μl emulsification consisting of spinal cord homogenate in phosphate buffered saline (PBS) (1:2; *v*/*w*) and CFA (1:1; *v*/*v*), at the tail root. Previous studies have shown that this immunization regimen results in 100 % disease induction [14, 15]. CFA controls were immunized with the same amount of emulsion without the spinal cord homogenate, whereas the PBS animals were injected with PBS only. During this procedure, the animals were anesthetized using 3 % isoflurane in a mixture of O_2_ and N_2_O (2:1). Immunizations were conducted completely randomized in order to minimize cage, experimental, and seasonal variability.

### Experimental groups

A total of eight experimental groups were included in the study. One PBS group (*n* = 6) sacrificed 5 days post-injection; three CFA groups sacrificed 3 (CFA 3, *n* = 6), 5 (CFA 5, *n* = 8), and 7 (CFA 7, *n* = 8) days post-immunization (DPI); three spinal cord homogenate + CFA groups sacrificed 3 (3 DPI, *n* = 8), 5 (5 DPI, *n* = 10), 7 (7 DPI, *n* = 10), and 12–13 (EAE, *n* = 6) DPI. EAE animals were scored on a daily basis for clinical disease symptoms according to the EAE clinical scoring system (0: no clinical symptoms to 6: moribund or dead) devised by the Danish Animal Experiments Inspectorate. When the scores reached 2, animals were provided with food and water-gel at the bottom of the cage. If the animals reached a score above 4, or if weight loss exceeded 20 %, the animals were euthanized.

### Tissue preparation

After the animal was removed from the stereotaxic frame, still under anesthesia, the femoral vein was exposed and a catheter inserted. Blood was withdrawn into heparinized micro-centrifuge tubes, centrifuged at 400×*g* for 8 min, and the aspirated plasma layer stored at −80 °C for later analysis. The cell pellet was resuspended in red blood cell lysis buffer (Biolegend, California, USA), incubated on ice for 5 min, centrifuged (400×*g*, 8 min), and frozen. The animals were perfused with 100 ml ice-cold PBS, pH = 7.2, and decapitated under deep isoflurane anesthesia.

One random brain hemisphere was frozen in isopentane and stored at −80 °C and the other processed for mitochondrial isolation (see below).

Pieces of tissue from the cervical, thoracic, and lumbar/sacral regions of the spinal cord were frozen in isopentane and stored at −80 °C, processed for mitochondrial isolation, or frozen in isopentane at −80 °C, and embedded in Tissue-Tek® O.C.T. compound (Sakura Finetek, Alphen Aan Den Rijn, The Netherlands) for cutting. The right liver lobe was frozen in isopentane and stored at −80 °C. The optic nerve was frozen in isopentane at −80 °C and embedded in Tissue-Tek® O.C.T. compound (Sakura Finetek) for sectioning.

### Quantification of mitochondrial membrane potential

No more than 1 h after isolation, the brain and spinal cord were processed for mitochondrial isolation and analysis. In a Dounce homogenizer, the cells were gently homogenized in DMEM (Thermo Fisher Scientific, Massachusetts, USA) and the mitochondria were isolated with differential centrifugation before resuspension in respiration buffer (according to the manufacturer’s instructions, Mitochondria isolation kit, Sigma-Aldrich). The mitochondrial suspension was processed immediately for quantification of membrane potential or stored at −80 °C for later analysis. The membrane potential in the suspension was quantified with the lipophilic cationic fluorescent dye JC-1 (Sigma-Aldrich). The dye accumulates in the mitochondrial matrix as a function of the electrochemical gradient across the inner mitochondrial membrane. When excited at 490 nm, the emission spectrum is dependent on the concentration of JC-1. At higher concentrations, aggregates will form and the fluorophore will emit at 590 nm, whereas dilute solutions will result in emission at 527 nm. After addition of JC-1, the samples were incubated 10 min before measuring fluorescent intensity every 5 min for 60 min on a Fluostar Optima (BMG Labtech, Offenburg, Germany). The 590:525 nm ratio was used to assess the mitochondrial membrane potential where a decrease indicates mitochondrial depolarization. Control runs were always performed to test the specificity of the assay, by the addition of valinomycin, a K^+^-specific transporter which results in complete dissipation of the membrane potential (Sigma-Aldrich).

### Immunohistochemistry

The embedded spinal cord and optic nerve were cut into 10-μm sections on a Leica CM 3000 cryostat (Leica Microsystems, Wetzlar, Germany). For detection of 8-hydroxydeoxyguanosine (8-OHdG), a modified base generated due to reactions with hydroxyl radicals, the tissue sections were fixed in acetone for 2 × 10 min. This was followed by incubation in 2 M HCl for 10 min to denature the double-stranded helix and then proteinase K treatment (10 μg/ml) for 8 min to remove DNA-associated proteins. After blocking in 5 % goat serum, the sections were incubated with the anti-8-OHdG antibody (1:300, Abcam, Cambridge, UK) overnight at 4 °C. Following incubation with chromeo 546-conjugated goat anti-mouse antibody (1:600) for 1 h, the sections were mounted with Vectashield mounting medium with DAPI (Vector Labs, Burlingame, California, USA). Positive “spots” in two representative areas each from the white and gray matter in the spinal cord, as well as the entire optic nerve cross section were identified. They were given a score from 1 to 3 according to size, and the total sum is given for each group. Only spots coinciding with DAPI staining (about 90 %) were included in the quantification. The counting was performed blinded using the Axiovert 200M LSM 520 and the software LSM 510 META (Carl Zeiss, Oberkochen, Germany). Spinal cord cross sections were furthermore stained with Luxol fast blue and cresyl violet in order to detect demyelination. Briefly, 16 sections from each of the PBS, CFA 7, 3 dpi, 7 dpi, and EAE animals were stained overnight at 57 °C in 0.1 % Luxol fast blue. After washing, the staining was differentiated in 0.05 % lithium carbonate for 30 s, followed by washing and staining in 0.1 % cresyl violet for 10 min. After differentiation in 95 % ethanol, the sections were mounted in CC/Mount (Sigma-Aldrich) and the staining was analyzed using a Leica DMRBE microscope (Leica Microsystems). In addition, 16 sections from each of the same groups as mentioned above were stained with anti-CD3 antibody (1:100, Abcam). Briefly, the sections were blocked with 5 % goat serum for 30 min, followed by incubation overnight with the primary antibody. After washing, the sections were incubated with biotinylated goat anti-rabbit antibody (1:500, Dako, Glostrup, Denmark) for 1 h, followed by washing and incubation with ABC complex (avidin/biotin-peroxidase, Vectorlabs, California, USA) according to the manufacturer’s instructions. After thorough washing, incubation with DAB for 5 min was followed by mounting in CC/Mount (Sigma-Aldrich). Identification of CD3^+^ cells was performed blinded on a Leica DMRBE microscope (Leica Microsystems).

### Semi-quantitative determination of reactive oxygen/nitrogen species-induced degeneration

For western blotting, the brain, spinal cord, and liver were homogenized in RIPA buffer (200 mg tissue/ml buffer: 25 mM Tris HCl, pH 7.6; 150 mM NaCl; 1 % NP-40; 1 % sodium deoxycholate; 0.1 % SDS) including a cocktail of protease inhibitors (Complete mini, Roche, Penzberg, Germany) using a Dounce homogenizer. After centrifugation (2 × 12.000×*g*, 20 min), total protein determination (660 nm protein assay, Thermo Fisher Scientific, Massachusetts, USA) was performed followed by polyacrylamide gel electrophoresis and transfer onto PVDF (Thermo Fisher Scientific) membranes. After blocking, the membranes were probed with antibodies against carbonylated amino acid side chains (Oxyblot detection kit, Millipore, Massachusetts, USA), nitrated tyrosine residues (1:1000, Abcam), cleaved caspase 3 (17/19 kDa, 1:1000, Cell Signaling Technology (CST), Massachusetts, USA), cleaved caspase 9 (38/40 kDa, 1:1000, CST), cleaved PARP (89 kDa, 1:1000, CST), calpain-1 large subunit (75/80 kDa, CST), cleaved alpha-II spectrin (150/120 kDa, 1:1000, CST), non-phosphorylated Neurofilament Heavy (NF-H, 200/180 kDa, 1:1000, Abcam), and α-tubulin (1:2500, Abcam). Primary antibody incubations were followed by HRP-coupled anti-IgG antibody (anti-mouse and anti-rabbit, 1:20,000, Jackson Immunoresearch, Pennsylvania, USA) incubation, ECL (Thermo Fisher Scientific) incubation, and development on CL-XPosure films (Thermo Fisher Scientific). Band or whole lane intensity was quantified using the software ImageJ, and data normalized to α-tubulin.

### Quantification of cytochrome C, neurofilament-H, and chemokine/cytokine production

Determination of cytochrome C in cytoplasmic and mitochondrial fractions was performed after mitochondrial isolation from the brain and spinal cord according to the manufacturer’s instructions (Profiling ELISA kit, Abcam).

Determination of NF-H levels in the plasma was performed using ELISA (USBio, Massachusetts, USA), according to the manufacturer’s instructions.

Determination of chemokine/cytokine levels in the plasma and brain was done using the Bioplex multiplex system (Biorad, California, USA) according to the manufacturer’s instructions.

### Statistical analyses

590:520 nm ratios are given as mean values and compared using the Kruskal-Wallis test followed by Dunn’s multiple comparison test. Data obtained from the semi-quantitative determinations of whole lane or specific band intensity in western blots are given as mean ± SEM and compared using one-way ANOVA followed by Bonferroni’s multiple comparisons test. 8-OHdG intensity in nuclei in optic nerve cross sections are given as mean ± SEM and compared using Kruskal-Wallis followed by Dunn’s multiple comparisons test. Cytokine/chemokine, NF-H, and cytochrome C concentrations are given as mean ± SEM and compared using one-way ANOVA followed by Bonferroni’s multiple comparisons test. A number of T cells and neutrophil granulocytes in the peripheral blood and brain are compared using one-way ANOVA followed by Bonferroni’s multiple comparisons test. *p* < 0.05 was considered statistically significant.

## Results

### Disease characteristics

**Fig. 1:**
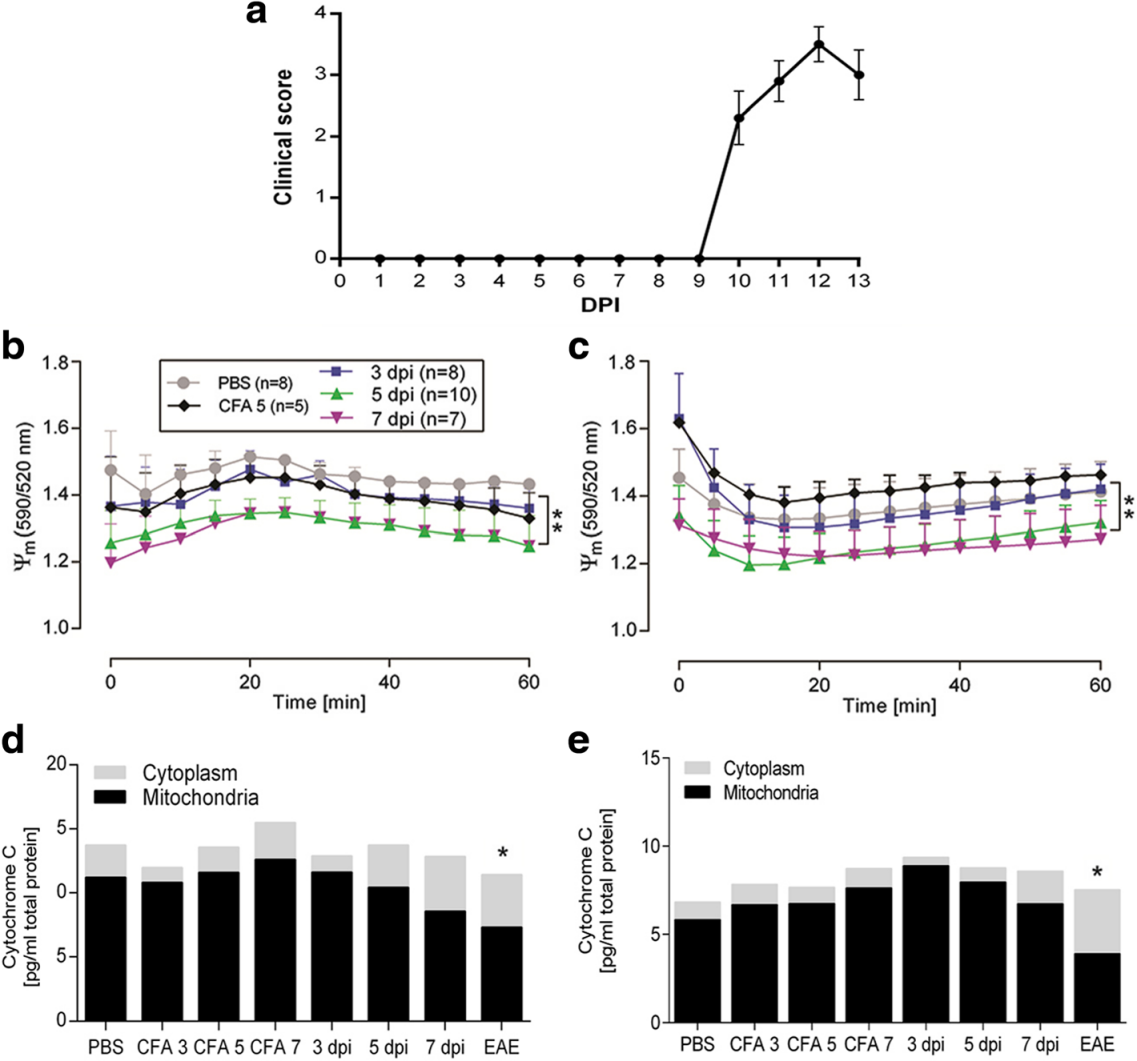
Disease curve of the EAE animals and determination of Δ^ψ^_m_ and cytochrome C release in the brain and spinal cord. The EAE animals were sacrificed between days 11 and 13, all with a clinical score ≥3 (**a**). The mitochondrial membrane potential was monitored continuously every 3 min, for 60 min, in the brain (**b**) and spinal cord (**c**) isolates. The ratio was significantly higher in the PBS and the CFA 5 groups compared to the 5 and 7 DPI groups in both the brain and spinal cord (mean ± SEM, (*p* < 0.01). Loss of cytochrome C from the mitochondrial to the cytoplasmic fraction in the brain (**d**) and spinal cord (**e**) was only significant in the EAE group, whereas the 7 dpi group showed a tendency (*p* < 0.059) towards decreased amounts in the mitochondrial fraction, compared to the CFA 7 group. *n =* 5 in each group in **d** and **e** (data are presented as mean ± SEM, *p < 0.05, **p < 0.01)

Earlier studies in our group have shown 100 % disease incidence following immunization with spinal cord homogenate emulsified in CFA [16, 17]. In this study, we also observed 100 % disease induction in the EAE group (mean day of disease debut 9.75 ± 0.89; mean relapse peak 12.50 ± 0.76; mean clinical score 3.50 ± 0.75, Fig. 1a).

### Mitochondrial degeneration

It is firmly established that irreversible demise of neural cells, following exposure to various insults, is linked to a decrease in the potential across the inner mitochondrial membrane (Δ^ψ^_m_) [7, 18, 19]. This typically coincides with the release of cytochrome C from the mitochondrial intramembrane space to the cytoplasm resulting in apoptosome formation, activation of caspase 9, and induction of apoptosis [20]. Therefore, we quantified Δ^ψ^_m_ and cytochrome C in brain and spinal cord tissue from the animals (Fig. 1b–e).

No differences in Δ^ψ^_m_ were found between PBS, CFA 5, and the 3 DPI animals, whereas a significant decrease—15–19 % reduction in area under the curve—was observed in the brain at days 5 and 7 post-immunization (Fig. 1b) and in the spinal cord at 7 days after immunization (Fig. 1c). This depolarization across the inner membrane could indicate that the electron transport chain is compromised or that the outer membrane is leaky, causing proton loss. Thus, we furthermore monitored cytochrome C release to the cytoplasm (Fig. 1d, e). Significant loss of cytochrome C from the mitochondria was only evident after disease debut in the EAE animals, although we observed a tendency at days 5 and 7 after immunization.

### Oxidative changes in the brain, spinal cord, and optic nerve

**Fig. 2:**
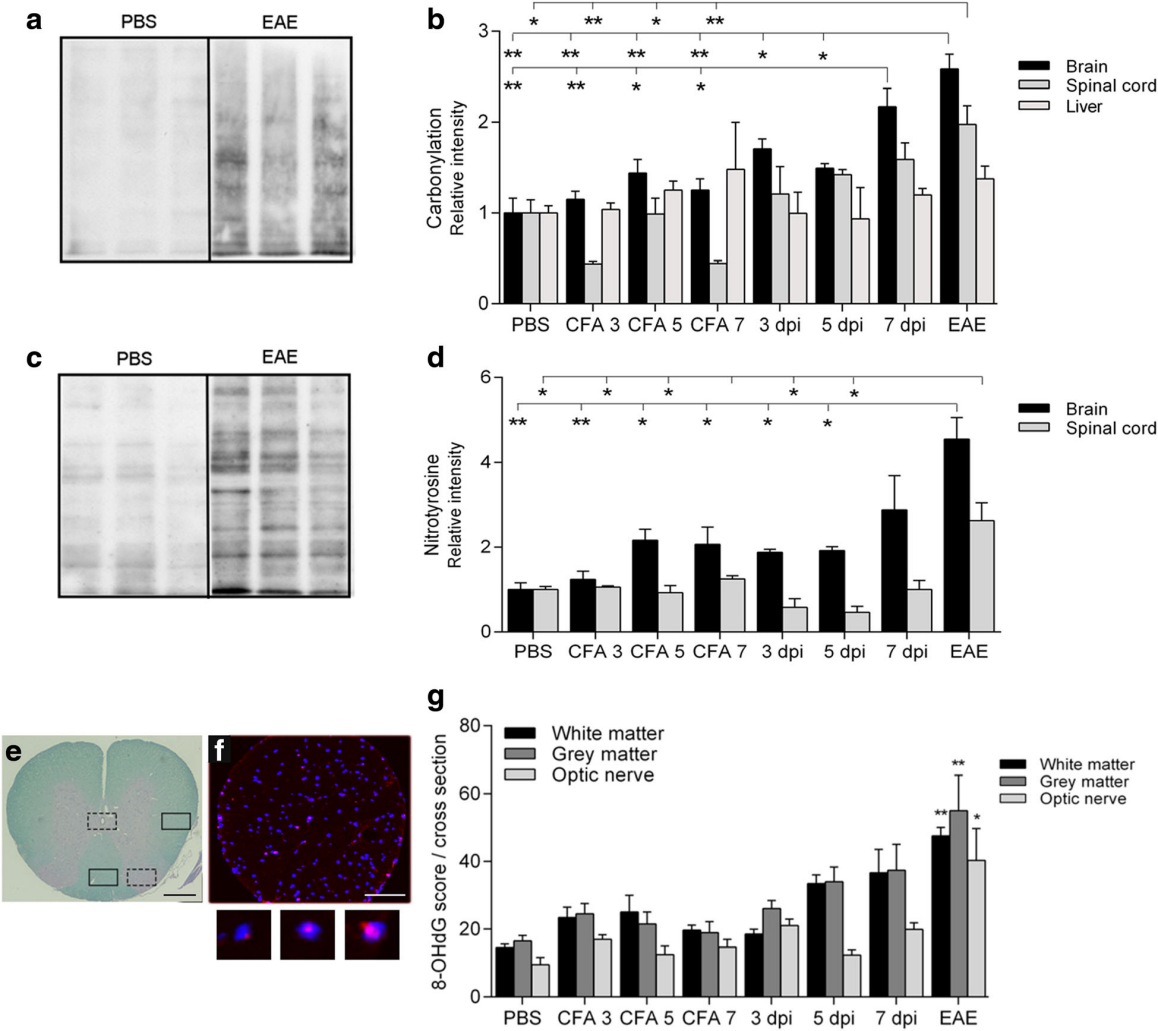
Semi-quantitative determination of oxidative stress in the brain, spinal cord, and optic nerve. **b** Semi-quantification of whole lane carbonylation (mean ± SEM) in the brain (*n =* 6–7/group), spinal cord (*n =* 6–8/group), and liver (reference tissue, *n =* 4/group), as exemplified in **a**. **d** Semi-quantitative determination of whole lane nitrosylation (mean ± SEM) in the brain (*n =* 5/group) and spinal cord (*n =* 5/group), as exemplified in **c**. **g** Mean score ± SEM of 8-OHdG positivity in spinal cord gray matter sections (*n =* 14–50/group) white matter sections (*n =* 14–50/group, quantified regions are shown in **e**, *scale bar* 500 μm), and optic nerve sections (*n =* 14–40/group), exemplified in **f** (8-OHdG: *red*, DAPI: *blue*, *scale bar* 50 μm). Examples of 8-OHdG positive “spots” scored 1, 2, and 3, respectively, are shown below. A significant upregulation of protein carbonylation in the brain was seen already 7 days after immunization, whereas tyrosine nitration and formation of 8-OHdG was increased in diseased animals only. Immunization had no impact on protein oxidation in liver tissue. *p < 0.05, **p < 0.01, ***p < 0.001

It is known that disturbances in Δ^ψ^_m_ are intimately involved in redox homeostasis within the cell [21]. Therefore, we next monitored the level of oxidation in snap-frozen homogenates from the brain and spinal cord as well as cryosectioned optic nerves. We did that by estimating the amount of protein carbonylation as a marker for reactive oxygen species-induced modifications of amino acid side chains, nitrotyrosine as a marker for peroxynitrite-mediated nitration, and 8-OHdG as a marker for oxidative DNA damage (Fig. 2).

A significant increase in the amount of carbonylated proteins was seen in brain tissue from 7 days after immunization (2.2-fold) and EAE animals (2.6-fold) (Fig. 2b). This coincides with the early decrease in Δ^ψ^_m_, probably reflecting reactive oxygen species-induced damage to components of the electron transport chain [22]. In contrast, the nitration of tyrosine residues was increased in EAE animals only (4.6-fold, Fig. 2d).

Noteworthy amounts of 8-OHdG, a modified base that serves as a marker for oxidative modifications in DNA induced by the hydroxyl radical (^•^OH), were seen in all groups, probably owing to atmospheric exposure (Fig. 2g). A tendency towards an increase was seen in the spinal cord from the 7 DPI animals (*p* < 0.078), whereas significant increases were seen in both the spinal cord and optic nerve in EAE (~2.6-fold upregulation compared to the CFA 7 animals). Thus, oxidative stress seems to be involved in the earliest neurodegenerative events, primarily affecting the brain.

### Degeneration and cell death

**Fig. 3:**
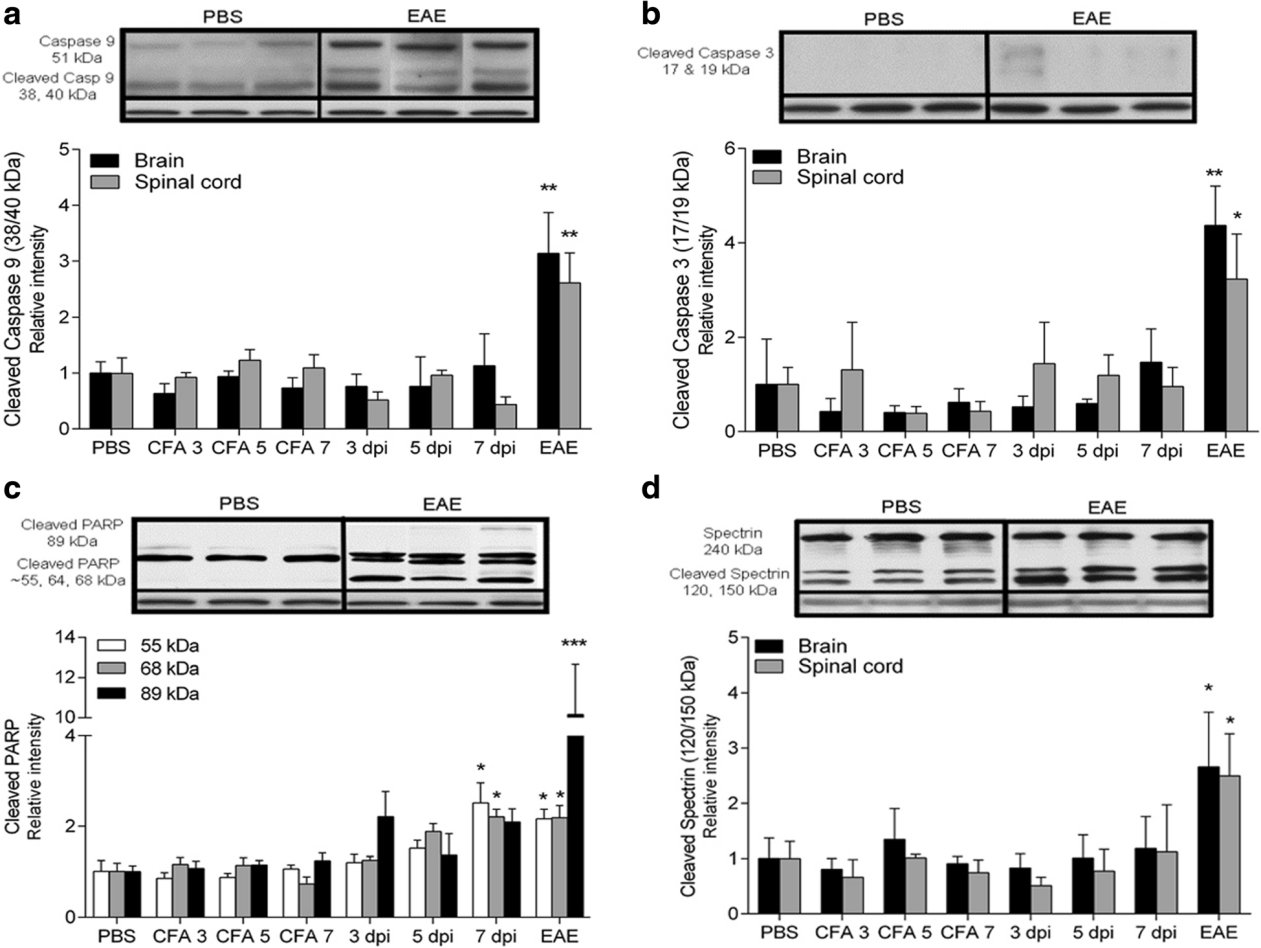
Semi-quantitative determination of cleaved caspase 9, cleaved caspase 3, cleaved PARP, and cleaved alpha-II spectrin in the brain and spinal cord. The apoptotic markers cleaved caspase 9 (**a**, *n =* 6/group), cleaved caspase 3 (**b**, *n =* 6/group), and cleaved PARP (89 kDa, **c**, *n =* 6/group) are increased in EAE animals only. The N-terminal PARP fragments (55 and 68 kDa, **c**) are upregulated in 7 DPI animals also. Cleaved alpha-II spectrin (**d**, *n =* 6/group) is increased in EAE animals only. α-Tubulin is included as loading control. Mean ± SEM. *p < 0.05, **p < 0.01, ***p < 0.001 compared to CFA 7 animals

It is known that mitochondria are intimately involved in the control of apoptotic pathways in neural degeneration [7, 19, 23]. Therefore, we went on to investigate whether the mitochondrial depolarization and the increased oxidative stress early after immunization could result in activation of apoptotic and/or necrotic markers. We probed the membranes with antibodies against cleaved caspase 9 (40 and 38 kDa), cleaved caspase 3 (19 and 17 kDa), and cleaved PARP (89 and N-terminal fragments from ~70–50 kDa), as well as cleaved alpha-II spectrin (150 and 120 kDa) (Fig. 3).

Neither caspase 9, caspase 3, nor PARP (89 kDa) showed evidence of apoptotic activity before disease was evident (Fig. 3a–c). Thus, the attenuated Δ^ψ^_m_ observed at pre-EAE time-points, with probable dire consequences for the bio-energetic function of the organelle, does not seem to be related to cytochrome C release with activation of caspases 9 and 3 and subsequent 89 kDa PARP fragmentation. In EAE animals, the apoptotic machinery is activated both in the brain and spinal cord due to both neural and leukocytic death. However, cleavage of PARP into several alternative fragments at around 55, 64, and 68 kDa occurred in the 7 DPI animals as well as the EAE animals (Fig. 3c). Proteases other than caspase 3 are known to cleave PARP into fragments of various sizes, and the pattern observed here could indicate calpain-1 activity [24]. This, together with the fact that a decrease in Δ^ψ^_m_ is known to be associated with necrotic cell death as well [25], prompted us to look at the cleavage pattern of the calpain-1 substrate alpha-II spectrin. However, spectrin cleavage into 120- and 150-kDa fragments, known to be present during apoptosis as well [26], was only observed in the diseased animals (Fig. 3d).

**Fig. 4:**
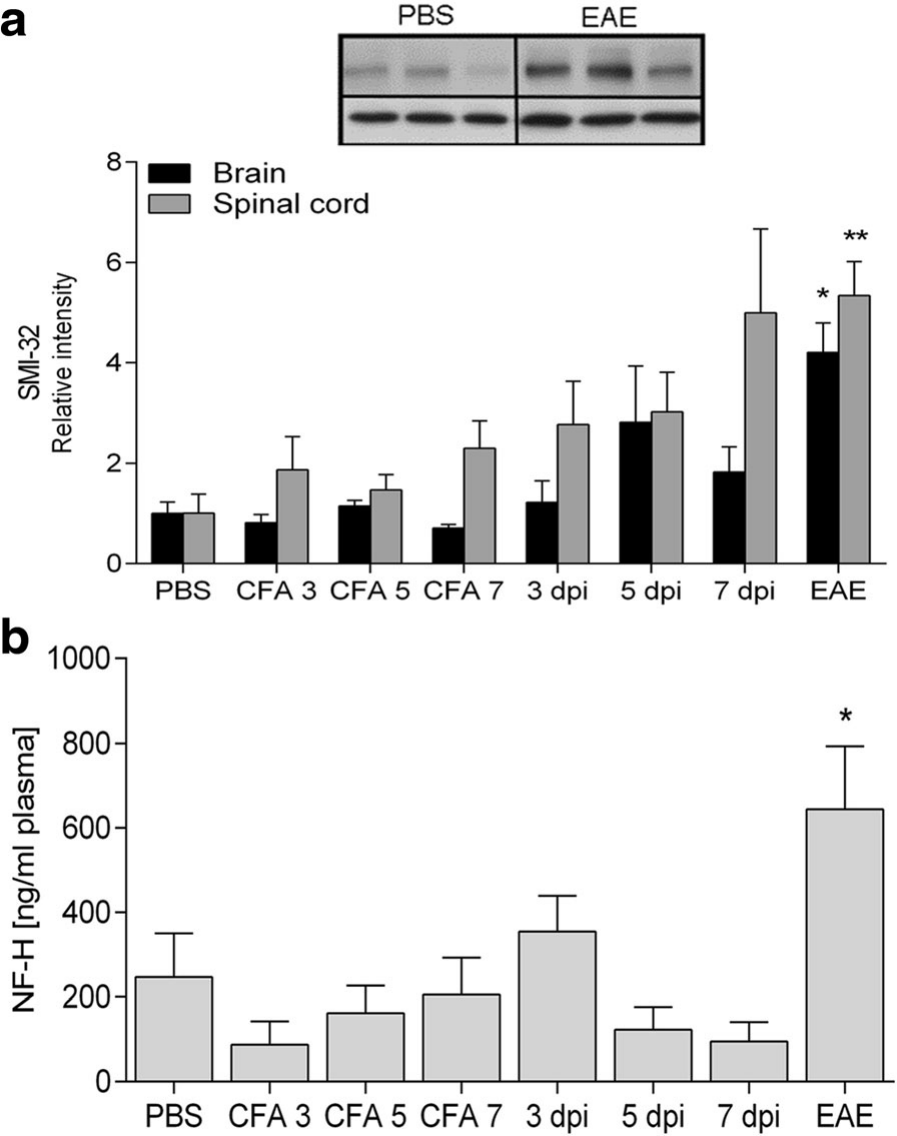
Levels of non-phosphorylated NF-H (SMI-32) in the brain (*n =* 6–9/group) and spinal cord (*n =* 6–8/group) and NF-H in the plasma (*n =* 6–10/group). Non-phosphorylated NF-H (**a**) as well as NF-H in the plasma (**b**) are increased in EAE animals only. α-Tubulin is included as loading control. Mean ± SEM. *p < 0.05, **p < 0.01, compared to the CFA 7 group

In order to clarify whether any neurodegenerative processes take place before inflammatory cell infiltration, we determined the levels of a dephosphorylated epitope in NF-H (Fig. 4a), shown to be associated with early axonal degeneration [27]. In addition, we also tested whether we could detect NF-H in the plasma as a marker of axonal destruction (Fig. 4b). However, degeneration and cleavage of axons, with subsequent release of axonal components to plasma, were only detectable in the EAE animals.

### Inflammatory responses in the brain and peripheral blood

**Fig. 5:**
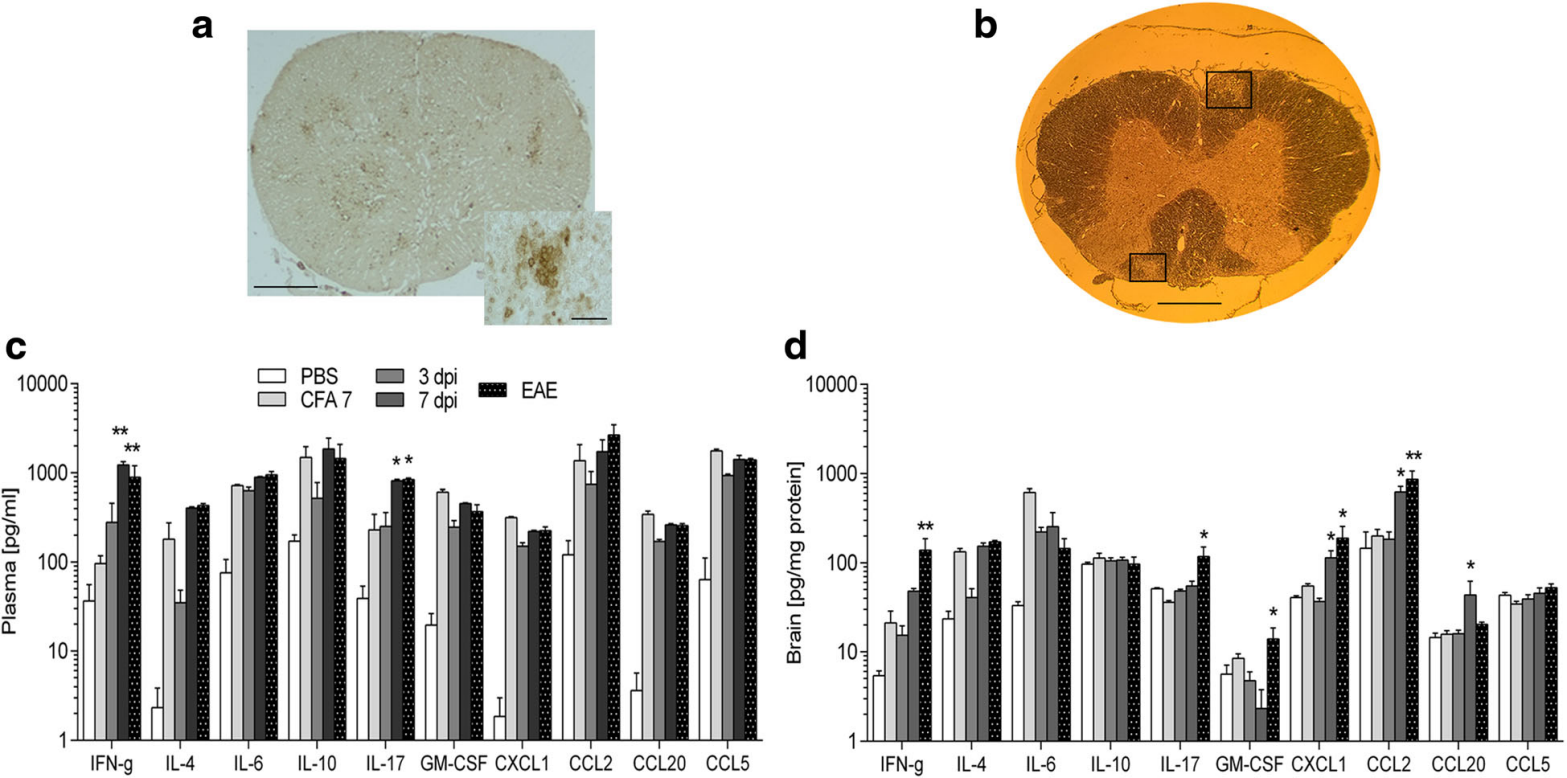
Inflammatory response in CNS tissue and peripheral blood. No signs of CD3^+^ T cell infiltration or demyelination were seen in pre-diseased animals, whereas the EAE animals had massive T cell infiltrates (**a**, *scale bar* 500 μm) and noduli (**a**, *insert*, *scale bar* 50 μm) and areas of demyelination (**b**, indicated by *two black rectangles*, *scale bar* 500 μm). Concentrations of a wide variety of cytokines and chemokines in the plasma (**c**, *n =* 5–6/group) and brain (**d**, *n =* 6/group). In the plasma, a general upregulation was seen as a consequence of immunization with CFA, compared to PBS, whereas IFN-γ and IL-17 was increased further if spinal cord homogenate was present (7 dpi and EAE animals). In the brain, the chemokines *Cxcl1*, *Ccl2*, and *Ccl20* were all increased in the 7 dpi animals, whereas IFN-γ and IL-17 were increased in the EAE animals, compared to the CFA 7 group. Mean ± SEM. *p < 0.05, **p < 0.01, and ***p < 0.0001 indicates data significantly different from the CFA 7 animals

Next we wanted to investigate whether an inflammatory response in the CNS could be a causal factor behind the subtle degenerations seen at day 7 post-immunization. Presence of T cells (Fig. 5a) and a panel of chemokines/cytokines (Fig. 5c, d) were analyzed at early time-points after immunization. We furthermore stained for myelin in spinal cord cross sections (Fig. 5b) to determine whether any early demyelination was occurring.

We were not able to detect any CD3^+^ T cells in spinal cord tissue before disease was evident clinically (Fig 5a). Furthermore, no demyelination was detected in pre-disease animals (Fig. 5b).

Given that no significant CNS influx of immune cells occurs at 7 dpi, the mitochondrial (Fig. 1b) and oxidative (Fig. 2b) alterations observed are probably caused by factors unrelated to direct immune cell cytotoxicity.

In the plasma, the concentration of several chemokines/cytokines significantly increased as a consequence of immunization with both CFA and with spinal cord homogenate + CFA, compared to the PBS group (Fig. 5c). With regard to IFN-γ and IL-17, the increase was potentiated when spinal cord homogenate was present, pointing towards their role as effector cytokines in EAE. IFN-γ increased from 137 pg/ml in the CFA 7 animals to 1302 pg/ml in the 7 DPI animals and 890 pg/ml in the EAE animals. IL-17 went from 50 pg/ml in the CFA 7 animals to 816 pg/ml in the 7 DPI animals and 836 pg/ml in the EAE animals.

In the brain, a more tightly regulated response was seen (Fig. 5d), especially with regard to the chemokines *Cxcl1*, *Ccl2*, and *Ccl20*, which were highly upregulated already in the 7 DPI animals, indicating that specific homing to the CNS occurs after administration of an encephalitogenic emulsion. At this time-point, no or very few inflammatory cells have infiltrated the CNS, thus signifying that these chemoattractants are produced by non-infiltrating immune cells very early during disease development, as indicated by a previous study [28]. Furthermore, significantly increased levels of IFN-γ, IL-17, and GM-CSF were seen in the EAE group (Fig. 5d), as shown previously [23].

## Discussion

This study investigated the significance of a range of degenerative processes including changes in mitochondrial membrane potentials, redox levels, and cell death markers, as well as T cells and mediators in brain, spinal cord, and optic nerve tissue, in pre-disease stages of EAE. We found subtle decreases in the mitochondrial membrane potential, coupled with an increased reactive oxygen species burden in brain tissue at 5–7 days after immunization. This was associated with a specific expression pattern involving the chemokines *Ccl2*, *Ccl20*, and *Cxcl1*. Degenerative processes at late time-points included increased oxidative stress and PARP fragmentation at day 7 after immunization, but these events did not trigger caspase-mediated or necrotic cell death. Thus, neural degeneration and cell death are either absent or below our threshold of detection, before disease activity is present.

Early degenerative changes specifically related to mitochondria in EAE have been reported, and this organelle is known to be involved in both early and late phases of cell death [19, 25]. Already 3 days after immunization, investigators have observed reductions in Δ^ψ^_m_ and increases in both nitrative stress and apoptosis [7, 8]. However, they used the cationic and lipophilic fluorescent probe Mitotracker Red to monitor Δ^ψ^_m_, which may exhibit different properties compared to JC-1. Data indicate that Mitotracker Red is sensitive to Δ^ψ^_m_ in cultured neurons, but insensitive in astrocytes, and moreover seems sensitive to reactive oxygen species burden [29]. We did not detect differences in the levels of nitrated tyrosine residues before EAE onset, in mitochondrial isolates and whole tissue homogenates, in contrast to previous results by other investigators [7]. However, they detected very low levels of nitrotyrosine in the control animals, whereas we saw several nitrated bands in the PBS group (Figs. 1e and 2c). A possible explanation for this discrepancy is that peroxynitrite formation and subsequent nitration spontaneously occurred during the isolation procedure in respiration buffer containing ATP and succinate. Nitration of key mitochondrial proteins such as subunits of the mitochondrial respiratory chain complexes I and IV and mitochondrial heat shock protein 70 were identified in the Qi study [7]. The nitration and possible subsequent inactivation of these proteins could be causally linked to the decreased mitochondrial membrane potential we observed at 5 and 7 days post-immunization.

Our finding, that significant loss of cytochrome C from the mitochondria was evident only after disease debut in the EAE animals, indicates that activation of the intrinsic apoptotic pathway, and thereby execution of programmed cell death, occurs at a relatively late time-point where inflammatory cells have entered the CNS and disease activity is present.

Several reports have shown that axonal degeneration precedes inflammatory and demyelinating events in myelin-immunized EAE animals [6, 7, 9, 10]. Two studies by different investigators have shown axonal and neuronal loss at 5 and 7 days post-immunization, respectively [6, 10]. Both studies used manual counting of retinal ganglion cells in the medial dorsal column. These data differ from our findings, where cell death was non-detectable before EAE onset (Fig. 4). Instead of manual counting for detection of neuronal/axonal loss, we investigated whether caspase 9 or caspase 3 were activated, and furthermore if NF-H could be detected in serum as a sign of axonal degeneration [30, 31]. These investigations were negative in our model. Additionally, we did not find changes in the levels of oxidative DNA modifications in the optic nerve after immunization. Since the optic nerve consists of axons, running from neuronal cell bodies located in the retina, and glial cells, the nuclei present belong to non-neuronal cells and degenerative changes thus reflect glial cell damage.

Initially and before commencing this study, we speculated that early neural degenerations could be a consequence of cytokines being upregulated in the CNS following immunization. Both MS and EAE show peripheral and central immune activation orchestrated by a plethora of mediators [32]. Administration of LPS (Toll-like receptor 4 (TLR4) ligand) or immunization with an emulsion containing CFA causes a systemic immune activation with production of cytokines and chemokines. These mediators most likely interact with cells in the CNS, e.g., microglia, through the choroid plexus and/or by direct transport across the blood-brain barrier [28, 33–35]. CFA exerts various effects through stimulation of TLR2 by *Mycobacterium tuberculosis* present in the adjuvant [36]. TLR2 activation results in the production of a variety of different inflammatory mediators, possibly also in the circumventricular organs where TLR2^+^ microglia are present [34, 37]. CFA is furthermore known to compromise the integrity of the blood-brain barrier [34]. Together, this results in significant increases in the concentration of a variety of cytokines in CNS tissue. The presence of significant amounts of these soluble mediators, could also explain the atrophy and degenerative changes observed distant from inflammatory infiltrates in MS and EAE [35, 36].

As stated, we found increased expression of the chemokines *Ccl2*, *Ccl20*, and *Cxcl1* in brain tissue, early after disease induction. In 1995, Glabinsky et al. found that MCP-1 (*Ccl2*) was non-detectable if leukocyte infiltration was absent, indicating that this chemokine may enhance but never initiate inflammation [38]. This is in contrast to our current data and results from other recent investigations [13]. We have no solid explanation for such different findings, although it should be noted that Glabinsky et al. used a different model and measured mRNA levels instead of proteins.

In the choroid plexus, constitutive expression of *Ccl20* has been shown to act as a gateway for T cells into uninflamed CNS, and Th17 cells preferentially express CCR6, the receptor for *Ccl20* [28]. Thus expression of *Ccl20* in the CNS plays a critical role in the entry of pro-inflammatory Th17 cells into the CNS. *Cxcl1* is known to attract neutrophils and has in other EAE studies been observed to peak at pre-clinical stages [39, 40]. Furthermore, systemic levels of this chemokine correlate with clinical disease activity and lesion burden in MS patients [40].

## Conclusions

Several days before clinical disease was present, subtle decreases in mitochondrial membrane potential and increases in reactive oxygen species was observed in brain tissue. No cell death was detected at these time-points, but a specific chemokine expression pattern indicates early chemoattractant activity in the brain of immunized animals.

## Abbreviations

CFA: Complete Freund’s adjuvant
EAE: Experimental autoimmune encephalomyelitis
MS: Multiple sclerosis
PBS: Phosphate buffered saline
TLR: Toll-like receptor
